# An acute psychotic disorder revealing hyperthyroidism by thyroid neoplasia: A case study

**DOI:** 10.1192/j.eurpsy.2021.657

**Published:** 2021-08-13

**Authors:** S. Sellami, N. Halouani, A. Chamseddine, F. Ben Othman, J. Aloulou

**Affiliations:** 1 Psychiatrie, Hedi Chaker University hospital, Psychiatry, Sfax, Tunisia; 2 Psychiatry, Hedi Chaker University hospital, sfax, Tunisia; 3 Psychiatry, Hedi Chaker University hospital, Psychiatry, sfax, Tunisia; 4 Psychiatry, Hedi Chaker University hospital, Psychiatry, Sfax, Tunisia

**Keywords:** dysthyroidism, psychosis, tumor, hyperthyroidism

## Abstract

**Introduction:**

Rarely, thyroid cancer can lead to hyperthyroidism. The link between dysthyroidism and psychiatric symptoms is well established, but cases of psychosis associated with hyperthyroidism are rarely reported in the literature.

**Objectives:**

Identifying psychosis secondary to hyperthyroidism caused by a secreting tumor through a case and literature review.

**Methods:**

We report the case of a patient with thyroid suspect tumor and chronic psychosis. We performed a literature review based on a PubMed search with the following keywords: “dysthyroidism psychosis”.

**Results:**

Mr. S,32, with a personal psychiatric history of chronic psychosis evolving since 4 years, without notable pathological history, was hospitalized in psychiatry for psychomotor instability, verbal hetero-aggressiveness, subtotal insomnia and refusal of treatment. The psychiatric examination revealed the presence of a chronic delusional syndrome with a theme of persecution, mysticism,and an interpretive, intuitive and hallucinatory mechanism, without dissociative syndrome. The somatic examination objectified a cachectic patient with a bilateral symmetrical non-impulsive exophthalmos, a goiter with a thrill on palpation, dysphonia and sinus tachycardia.A laboratory workup revealed inflammatory syndrome, collapsed TSH (<0.05 mU / L) and an increased T4 to 37 pmol / L. Cervical ultrasound showed a strongly suspect left lobar heteronodular goiter and poorly structured peripheral lymphadenopathy (TI-RADS 4-B). Sedative diazepam therapy was started with antithyroid therapy and a beta blocker. The evolution was quickly favorable. The patient is referred for surgical treatement.

**Conclusions:**

The severity of the hyperthyroidism,neoplastic origin, the improvement in psychotic signs with antithyroid treatment are arguments in favor of the thyroid origin by thyroid neoplasia.

